# Adjuvant therapy with high dose vitamin D following primary treatment of melanoma at high risk of recurrence: a placebo controlled randomised phase II trial (ANZMTG 02.09 Mel-D)

**DOI:** 10.1186/1471-2407-14-780

**Published:** 2014-10-24

**Authors:** Robyn PM Saw, Bruce K Armstrong, Rebecca S Mason, Rachael L Morton, Kerwin F Shannon, Andrew J Spillane, Jonathan R Stretch, John F Thompson

**Affiliations:** Melanoma Institute Australia, Poche Centre, 40 Rocklands Road, North Sydney, NSW 2060 Australia; Central Clinical School, Sydney Medical School, The University of Sydney, Sydney, Australia; Division of Surgery, Royal Prince Alfred Hospital, Sydney, Australia; Sydney School of Public Health, The University of Sydney, Sydney, Australia; Physiology and Bosch Institute, School of Medical Sciences, The University of Sydney, Sydney, Australia; Australia and New Zealand Melanoma Trials Group (ANZMTG), Poche Centre, North Sydney, Australia; Northern Clinical School, Sydney Medical School, The University of Sydney, Sydney, Australia

**Keywords:** Melanoma, Vitamin D, Randomised trial, Safety, Toxicity, Recurrence, Recruitment

## Abstract

**Background:**

Patients with primary cutaneous melanomas that are ulcerated and >2 mm in thickness, >4 mm in thickness and those with nodal micrometastases at diagnosis, have few options for adjuvant treatment. Recent studies have suggested a role for vitamin D to delay melanoma recurrence and improve overall prognosis.

**Methods/Design:**

This is a pilot placebo-controlled randomised phase II trial to assess the feasibility, safety and toxicity of an oral loading dose of Vitamin D (500,000 IU) followed by an oral dose of 50,000 IU of Vitamin D monthly for 2 years in patients who have been treated for cutaneous melanoma by wide excision of the primary. Patients aged 18 – 79 years who have completed primary surgical treatment and have Stage IIb, IIc, IIIa (N1a, N2a) or IIIb (N1a, N2a) disease are eligible for randomisation 2:1 to active treatment or placebo. The primary endpoints are sufficiency of dose, adherence to study medication and safety of the drug. The secondary endpoints are participation and progression free survival. The study has been approved by the Ethics Review Committee (RPAH Zone) of the Sydney Local Health District, protocol number X09-0138.

**Discussion:**

Effective, non-toxic adjuvant therapy for high risk primary melanoma is not currently available. Favorable outcomes of this phase II study will form the basis for a multi-centre phase III study to assess whether the addition of oral high-dose vitamin D therapy in patients who have completed primary treatment for melanoma and are at high risk of recurrence will:

1. prolong time to recurrence within 5 years
2. improve overall survival at 5 years and
3. be both safe and tolerable.

62 patients have been randomised since the study commenced in December 2010. Target accrual for the study has been met with 75 patients randomised between December 2010 and August 2014.

The Mel-D trial is conducted by the Australia and New Zealand Melanoma Trials Group (ANZMTG 02.09)

**Trial registration:**

Australia and New Zealand Clinical Trials Registry (ANZCTR) ACTRN12609000351213

## Background

Cutaneous melanoma (CM) is the fourth most commonly diagnosed cancer in Australia, with an incidence of 61.7 cases per 100,000 men, and 40.0 cases per 100,000 women in 2009. There are more than 12,500 new cases of melanoma diagnosed in Australia every year and the diagnosis rates have doubled in the past 20 years. CM is the sixth most common cancer in the United States, with an incidence of 21.3 per 100,000 per year (with an incidence of 35.8 cases per 1000,000 in men and 24.5 cases per 100,000 in women in 2011) [1]. Melanoma makes up only 2.3% of all skin cancers but is responsible for 75% of skin cancer deaths. It is also the most common form of cancer for people aged 15 to 44 years in Australia and accounts for more cancer related deaths in 20–34 year-olds Australians than any other cancer [2].

Since the mid-1960s, CM incidence has risen 3-8% per year in people of European background, with the greatest increases in elderly men [3]. Although there has been progressive improvement in 5 year survival, which is now greater than 85%, CM causes disproportionate mortality in those of young and middle age. Patients with ulcerated tumors thicker than 2 mm, with tumors thicker than 4 mm or with nodal micro-metastases at diagnosis (AJCC Stages IIb, IIc, IIIa (N1a, N2a) and IIIb (N1a, N2a); Tables 1 and 2) have a relatively poor prognosis with no known effective non-toxic adjuvant treatment available.

**Table 1:** **TNM classification for CM (3)**

Classification	Thickness (mm)		Ulceration status/Mitoses
Tis	N/A	a	N/A
T1			Without ulceration and mitosis <1/mm^2^
		b	With ulceration or mitosis ≥1/mm^2^
T2	1.01-2.00	a	Without ulceration
		b	With ulceration
T3	2.01-4.00	a	Without ulceration
		b	With ulceration
T4	>4.00	a	Without ulceration
		b	With ulceration
N	No. metastatic nodes		Nodal metastatic burden
N0	0	a	N/A
N1	1 node involved		Micrometastases*
	1 node involved	b	Macrometastases†
N2	2-3 nodes involved	a	Micrometastases
	2-3 nodes involved	b	Macrometastases
		c	Intransit metastases/ satellites without metastatic nodes
N3	4+ metastatic nodes or matted nodes or intransit metastases/satellites with metastatic nodes		
M	Site		Serum LDH
M0	No distant metastases		N/A
M1a	Distant skin, subcutaneous or nodal metastases		Normal
M1b	Lung metastases		Normal
M1c	All other visceral metastases		Normal
	Any distant metastasis		Elevated

*Micrometastases are diagnosed after sentinel lymph node biopsy.

†Macrometastases are defined as clinically detectable nodal metastases confirmed pathologically.

**Table 2:** **AJCC pathological staging system for CM (3)**

Stage	Primary tumour thickness and ulceration	Lymph node status	Distant metastasis
Stage 0	Tis	N0	M0
Stage IA	T1a	N0	M0
Stage IB	T1b	N0	M0
	T2a	N0	M0
Stage IIA	T2b	N0	M0
	T3a	N0	M0
Stage IIB	T3b	N0	M0
	T4a	N0	M0
Stage IIC	T4b	N0	M0
Stage IIIA	T1-4a	N1a	M0
	T1-4a	N2a	M0
Stage IIIB	T1-4b	N1a	M0
	T1-4b	N2a	M0
	T1-4a	N1b	M0
	T1-4a	N2b	M0
	T1-4a	N2c	M0
Stage IIIC	T1-4b	N1b	M0
	T1-4b	N2b	M0
	T1-4b	N2c	M0
	Any T	N3	M0
Stage IV	Any T	Any N	M1a
	Any T	Any N	M1b
	Any T	Any N	M1c

### Vitamin D

#### Vitamin D physiology

Vitamin D is a fat-soluble seco-steroid, which acts in the maintenance of calcium and phosphate homeostasis predominantly through increasing gut absorption of calcium and phosphate. Vitamin D is derived from 2 sources – endogenous (from synthesis in the skin – vitamin D_3_) and exogenous (dietary or supplements - vitamins D_2_ and D_3_). The substrate, 7-dehydrocholesterol (7-DHC), the penultimate compound in the cholesterol synthesis pathway, accumulates in the epidermis [4, 5]. Ultraviolet B radiation to the skin transforms 7-DHC to previtamin D_3_, which undergoes nonenzymatic isomerisation to form vitamin D_3_. Vitamin D_3_ is transferred into the blood stream by the vitamin D binding protein, an α‒globulin that has a high affinity to vitamin D and its metabolites.

**Figure 1:**
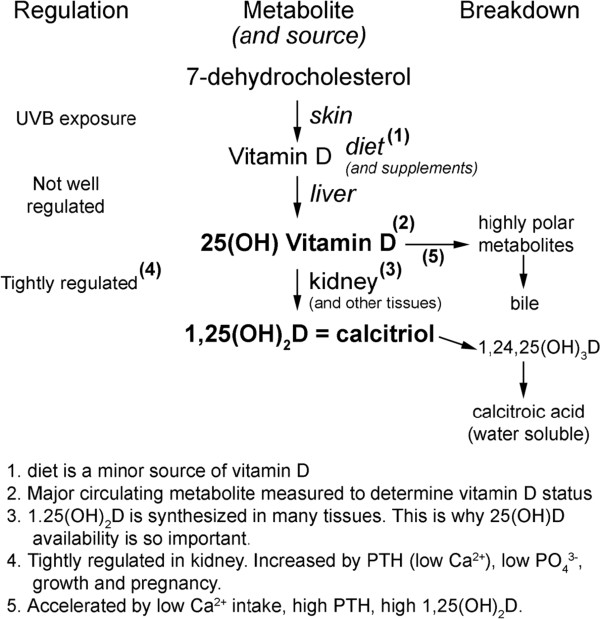
**Physiology of vitamin D.**

To be physiologically active, vitamin D (either D_2_ or D_3_) must first be hydroxylated to 25-hydroxyvitamin D (25OHD), predominantly in the liver and 25OHD then to 1α,25‒dihydroxyvitamin D (1α,25OHD), predominantly in the kidneys for export into the bloodstream, though many tissues, including skin, have the capacity to produce 1α,25OHD. Figure 1 shows the physiology of vitamin D in detail [6]. However, vitamin D can be activated by other pathways to metabolites which do not raise serum calcium [7].

#### Serum measurement of vitamin D

Serum 1α,25OHD has a very short half-life and is not a good measure of vitamin D status [8]. Serum 25OHD is the main circulating form of vitamin D. It is relatively stable [9], has a long half-life (τ1/2 = 18.9 +/- 3.1 days) [10] and provides the substrate for local production of the active hormone, 1α,25OHD and is therefore considered the best indicator of vitamin D status [11].

#### Vitamin D and mortality

A meta‒analysis of 18 randomised trials of supplementation with vitamin D provided evidence that supplemental doses in the range of 7.5 to 50 μg per day could significantly decrease all‒cause mortality. The summary relative risk for mortality from any cause was 0.93 (95% confidence interval, 0.87-0.99) [12]. The contribution of specific causes of mortality to the reduction in all‒cause mortality remains unknown.

#### Vitamin D and cancer including melanoma

1α,25OHD exerts physiological functions including regulation of growth and differentiation in a broad variety of normal and malignant cells [13–20] including melanoma [21]. The first study to report anti-melanoma activity by vitamin D showed in vitro, that 1α,25OHD inhibits cellular proliferation, and promotes differentiation and apoptosis, all properties compatible with antineoplastic action [22]. A recent study has also shown some novel hydroxyvitamin D analogues inhibit proliferation and colony formation of melanoma cells, similar to 1α,25OHD [7]. Slightly supraphysiological concentrations of 1α,25OHD have major effects on the cell cycle with a general but not complete G0/G1 cycle arrest [23].

Melanoma cells are able to convert the main circulating form of vitamin D, 25OHD, to 1α,25OHD [24], thus increasing the probability that vitamin D might be able to regulate their growth in vivo. This discovery has led to the hypothesis that autocrine or paracrine production of 1α,25OHD could prevent some cancers (e.g., prostate, colon, breast, pancreas, and ovary) and attenuate their progression. Together, these elements support the hypothesis that high serum 25OHD status could decrease the risk of cancer.

At least in part, because sun exposure, particularly intermittent exposure, is a risk factor for melanoma and sun exposure also increases vitamin D synthesis, there are no clear data relating 25OHD concentrations to risk of melanoma [25, 26]. However, there are additional observational data which supports the proposal that the vitamin D system or vitamin D status may affect the risk of melanoma and/or melanoma outcomes. Although the data are by no means entirely consistent, a recent meta-analysis showed that polymorphisms at two sites in the vitamin D receptor were associated with either reduced (Bsm 1 A allele) or increased (Fok 1 T allele) risk of melanoma [27]. Poorer outcomes from melanoma have been shown with melanoma cells that exhibit reduced or complete loss of vitamin D receptors or cytochrome P27B1 (CPY27B1 hydroxylates 25OHD) [28, 29]. Furthermore, in a post-hoc sub-group analysis of the Women’s Health Initiative randomized controlled trial, subjects with a history of non-melanoma skin cancer who were randomized to 1000 mg of elemental calcium and 400 IU of vitamin D per day had a reduced risk of melanoma (HR 0.43: CI0.1-0.90; P_interaction_ = 0.38) [30]. This was not seen in whole group analyses. In terms of outcomes, patients with melanomas surrounded by high solar elastosis [31] and those diagnosed in summer [32] show higher 5-year survival rates. Both of these factors could be related to higher prevailing 25OHD levels, but other interpretations are possible. Melanomas in patients with higher 25OHD concentrations around the time of diagnosis tend to have a lower Breslow thickness [27, 33], which is associated with a more favourable outcome. Furthermore, in observational studies of patients with melanoma, low 25OHD was associated with worse outcomes [34] and low 25OHD at the time of diagnosis was associated with an increased risk of progression and death over the subsequent 5 years [33]. While these findings are suggestive, the only way to determine whether increasing vitamin D status will improve melanoma outcomes is by conducting a randomized controlled trial [35]. However, several issues need to be addressed before a full scale trial is undertaken.

#### Raising 25OHD concentrations

Vitamin D supplements, usually in the form of vitamin D_3_, are used to raise 25OHD concentrations. When given at a standard dose of 25 or 50 μg (1000 or 2000 IU) per day, 25OHD concentrations take approximately 3 months to reach a plateau [36] and compliance with daily dosing is known to be problematic [37]. Bacon et al. suggested a dosing protocol of a loading dose of 12.5 mg (500,000 IU) of vitamin D_3_ followed by a monthly dose of 1.25 mg (50,000 IU) as a way to achieve an early increase in 25OHD concentrations and improve the likelihood of good compliance over the subsequent trial period, without causing hypercalcemia [38]. Although a high yearly dose of vitamin D of 12.5 mg in older women has been reported to be associated with an increase in falls over the subsequent 3 month period [39], no plausible mechanism has yet been determined, so that in the case of melanoma patients at high risk of recurrence, the possible benefits of this dosing regimen appear to outweigh the possible risks.

### Objectives of the present trial

To determine whether administration of a loading dose of oral vitamin D_3_ (500,000 IU) followed by a monthly tablet of 50,000 IU of oral vitamin D_3_ for 2 years following primary treatment of melanoma at high risk of recurrence:

1. achieves maintenance of serum 25 hydroxyvitamin D concentrations above 80 nmol/l in patients who receive active treatment
2. achieves > 80% adherence to dosing rates during 2 years of study treatment
3. achieves adequate recruitment rates (75 patients in 2 years from one site)
4. produces no clinically significant difference in hypercalcaemia incidence between the active and placebo groups
5. produces no clinically significant difference in renal function between the active and placebo groups
6. produces no clinically significant difference in the incidence of renal calculi between the active and placebo groups

#### Primary endpoints

The primary endpoints will be met if the following criteria are achieved:

1. Dose sufficiency
  If the majority of treated patients achieve a serum 25OHD of 80 nmol/l at both 12 and 24 months and the average serum 25OHD for treated patients is > 75 nmol/l at 12 and 24 months
2. Dose adherence
  If patients take > 80% of the prescribed monthly dose
3. Safety
  Calcium [40]
  ■ If the mean serum calcium concentration in each patient is <2.75 mmol/L (11 mg/dL) over the course of the study.
  ■ If there is no increase in the prevalence of hypercalcaemia relative to the baseline prevalence.
  ■ If the urine calcium excretion in each patient is less than 7.5 mmol/24 h OR the mean urinary calcium-creatinine ratio in each patient is <1.0 (when calcium and creatinine are measured in mmol) over the course of the study.
  ■ If there is no increase in the prevalence of hypercalciuria relative to the baseline prevalence.
  Kidney function
  ■ If there is no greater than a 20% fall in the average estimated GFR [GFR = 186.3 × SerumCr^-1.154^ × age^-0.203^ × 1.212 (if patient is black) × 0.742 (if female) – MDRD formula (21)] over the course of the study.
  ■ If the average eGFR at the end of the study is no more than 20% less in vitamin D treated patients than in placebo treated patients.
  Renal calculi
  If no more than two episodes of renal calculus occurs in vitamin D treated patients during the course of the study.

#### Secondary endpoints

The secondary endpoints will be met if the following criteria are achieved:

1. If 60% of eligible patients agree to participate and the study accrues 75 patients in 2 years
2. Improved progression free survival

#### Other tests

A sample of baseline blood will be stored for later extraction of DNA and possible testing for gene variants (e.g. in the vitamin D receptor gene or the vitamin D binding protein gene) that may influence vitamin D activity to see if they influence plasma vitamin D response to therapy or melanoma recurrence.

## Methods/Design

### Trial design

This is a multi-center placebo controlled randomised phase II trial which aims to evaluate the safety and efficacy of high dose vitamin D therapy in patients who have been treated for primary melanoma and are at high risk of recurrence. Patients will receive treatment at Melanoma Institute Australia (MIA) (at the Poche Centre or at the Royal Prince Alfred Hospital (RPAH)), or other participating melanoma study centres.

### Participants

Eligible participants are patients aged 18 – 79 who have completed primary (surgical) treatment for cutaneous melanoma and are considered to be at high risk for recurrence: AJCC Stage IIb, IIc, IIIa (N1a, N2a), IIIb (N1a, N2a), that is, patients with ulcerated tumors thicker than 2 mm or with tumors thicker than 4 mm or those with nodal micro-metastases. Comprehensive inclusion and exclusion criteria are found in Appendix 1.

### Intervention

- Materials
  Study medications will be provided under the Australian Therapeutic Goods Administration Clinical Trials Notification scheme. Patients randomised to the active treatment arm will receive Cal.D.Forte tablets containing 50,000 IU of cholecalciferol from API Consumer Brands New Zealand. Patients randomised to the placebo arm will receive placebo tablets from a batch prepared by API for an unrelated study and provided by that study’s coordinator, Dr Anna Ralph, of the Menzies School of Health Research, Darwin.
- Study drug administration
  Patients will receive oral vitamin D (Cal.D.Forte) tablets containing 50,000 IU of cholecalciferol from API Consumer Brands New Zealand or matching placebo.
  Following confirmation of eligibility and randomisation, patients randomized to active treatment will receive 500,000 IU of cholecalciferol (Vitamin D3) orally (10 tablets to be administered by the study team during the initial baseline study visit).
  Thereafter, patients will self-administer 50,000 IU, 1 Cal.D.Forte tablet on the 1st day of each month following commencement of treatment, for a period of 23 months.

### Assessment of outcome

- Dose sufficiency
  The short term effects of high dose vitamin D therapy will be assessed by conduct of the following assays and diagnostic tests:
  Renal and liver function tests (including estimated Glomerular Filtration Rate (eGFR) and urine calcium/creatinine ratio)
  Serum 25OHD levels
  Serum corrected calcium, 24 hour urinary calcium excretion or urinary calcium to creatinine ratio.
  The scheduling of these assessments is found in Table 1.
- Adherence
  Patients will be asked to keep a diary to record their use of the study drug. These diaries will be reviewed four monthly at each patient follow-up visit.
  The study team will confirm adherence to the treatment regime by contacting study patients at interim time points during the treatment phase and also during clinic follow-up visits at 4, 8, 12, 16, 20 and 24 months. To help improve adherence, monthly SMS text messages will be sent to those patients with mobile phones, reminding them to take their study tablets.
  In the event of a missed dose, patients will be able to take the missed dose at the following clinic visit. Appropriate procedures will be established to facilitate this while ensuring maintenance of necessary “blindness” with respect to the intervention arm each participant is in.
- Safety
  The feasibility, safety and short term effects of high dose vitamin D therapy will be assessed by the conduct of the assays and diagnostic tests found in Table 3.

**Table 3:** **Schedule of assessments**

	Baseline	4-6 wks	4 mth	8 mth	12 mth	16 mth	20 mth	24 mth
**History**	**X**		**X**	**X**	**X**	**X**	**X**	**X**
**Body mass index/height cm**	**X**		**X**	**X**	**X**	**X**	**X**	**X**
**Serum corrected calcium**	**X**	**X**	**X**	**X**	**X**	**X**	**X**	**X**
**Phosphate**	**X**	**X**	**X**	**X**	**X**	**X**	**X**	**X**
**24 hour urinary calcium (preferred)**	**X**	**X**	**X**					
**Urinary calcium/creatinine ratio** **(for first 3 time points only if 24 h urine unavailable)**	**X**	**X**	**X**	**X**	**X**	**X**	**X**	**X**
**Serum** **25O D**	**X**	**X**	**X**		**X**			**X**
**FBC**	**X**			**X**		**X**		**X**
**LFTs**	**X**			**X**		**X**		**X**
**eGFR**	**X**			**X**		**X**		**X**
**Adherence to dosing regime**	**X**		**X**	**X**	**X**	**X**	**X**	**X**
**Tumour status**	**X**		**X**	**X**	**X**	**X**	**X**	**X**

Note: Serum corrected calcium requires serum albumin.

An independent medical monitor with clinical expertise relevant to vitamin D and its effects has been appointed to adjudicate on all matters of patient safety. The monitor will be informed immediately if a result of any of the above tests is considered to be clinically significant (as determined by a local clinician) and will specify the action to be taken with respect to that result in the patient’s interest. Actions include dose modification, dose cessation and withdrawal from the trial because of low serum 25OHD concentration (see Appendix 2 for details).

### Sample size and randomisation

The design of the study is an optimal two-stage phase II trial using the approach of Simon [41]. Sample sizes are determined separately for the two cohorts. The two cohorts, those receiving vitamin D and those receiving placebo, will be analysed separately. Assuming a lower limit of efficacy of 48% and a regimen activity level of 68%, the study would proceed in two stages. In the first stage, if more than 7 patients with serum 25OHD less than 80 nmol/l are observed in the first 14 patients in the active arm, consideration will be given to either modifying the regimen or stopping the study due to inactivity.

Twenty-five patients (in a 2:1 randomisation) will be accrued to the placebo arm to allow adequate data to estimate concentration of serum 25OHD in an untreated cohort. Thus it is proposed to accrue a total of 75 patients into the study, 50 receiving vitamin D and 25 receiving placebo. These calculations are based on power of 80% with 95% confidence and allow for a modest dropout rate.

Patients will be centrally randomised by the Australian National Health and Medical Research Council (NHMRC) Clinical Trials Centre using a computerised interactive voice response system (IVRS). Patients will be stratified by gender.

### Accrual rate

The anticipated accrual rate is 75 patients in 2 years.

### Progression free survival

Analysis of the secondary endpoint of improved progression free survival will take into account patient stage subgroups. However, as the numbers in this study are small, a meaningful subgroup analysis may not be possible.

### Participant follow-up

Participation in this study is voluntary; patients will be able to withdraw at any time. Patients will be asked to present for clinical follow-up four monthly for the two years following treatment of their primary melanoma. Those who withdraw from treatment will be asked to continue follow-up visits according to the protocol even though they have stopped treatment, to allow collection of outcome data. If a patient decides to stop their follow-up visits, their health status will be periodically ascertained by way of phone contact with their general practitioner or by direct phone contact. The National Death Index at the Australian Institute of Health and Welfare will be used to collect survival information on patients who have been lost to follow-up.

After completion of active follow-up, recurrences will be ascertained through the New South Wales Central Cancer Registry and deaths through the National Death Index for a period of five years after date of diagnosis.

### Blinding

Patients and physicians are blinded to both study treatment allocation and vitamin D levels.

### Statistical methods

- Analysis of primary endpoints
  The percentage of patients achieving a serum 25OHD above 80 nmol/l at 12 and 24 months receiving treatment will be reported. Summary statistics of serum 25OHD concentrations will be reported for both groups at 12 and 24 months.
  Dose adherence will be reported as the percentage of dose taken compared to the prescribed monthly dose as calculated from the patient diary and study forms. A 95% confidence interval will be reported to check if the expected rate of 80% dosage of study tablets for the duration of the study protocol by all patients is obtained.
  Safety outcomes will be reported as the proportions of patients exceeding the following maxima, as well as summary statistics where appropriate.
- Analysis of secondary endpoints
  Participation rate will be reported as the proportion of subjects eligible and invited to participate who agree to be randomised. A 95% confidence interval will be reported to check if the expected rate of 60% was obtained.
  Progression free survival will be displayed using Kaplan-Meier survival curves.

### Quality assurance

An independent Data Safety Monitoring Committee will periodically review the study for efficacy and safety. Efficacy will be reviewed after the first 14 patients in the active arm have completed 4 and 12 months of the vitamin D regimen. Safety data will be reviewed after 20 patients have completed 4 and 12 months of treatment and again when 35 patients have completed 12 months of treatment.

## Discussion

### Feasibility

Initial accrual to the ANZMTG 02.09 Mel-D study was slow, however a number of strategies have recently worked well in the early identification of eligible patients. All histopathology reports from wide excisions of melanoma are checked by the clinical trials staff and potentially eligible study participants are identified. The melanoma surgeons are reminded about study eligibility criteria prior to that patient’s consultation. In addition, a one-page Mel-D trial synopsis detailing the inclusion and exclusion criteria has been placed in the surgical consulting rooms and frequent updates of the trial progress are reported at the MIA research meetings. The study is promoted regularly on the ANZMTG website, membership newsletter and at various meetings (including the Annual Scientific Research Meeting).

Approximately 50% of eligible patients have participated in the study. The target accrual of 75 patients has been achieved and 21 patients have completed the full 24 months of treatment and follow-up. However, 10 patients have developed recurrence and ceased the trial. Planning for a phase III trial is underway, to open to melanoma treatment sites outside the Melanoma Institute Australia.

### Registration

This trial is registered with the Australia and New Zealand Clinical Trials Registry (ANZCTR) #ACTRN12609000351213.

### Protocol

A full copy of the current ANZMTG 02.09 Mel-D protocol can be requested from the principal investigator Dr Robyn Saw, email: robyn.saw@melanoma.org.au.

### Appendix 1

#### Comprehensive inclusion and exclusion criteria for ANZMTG 02.09 Mel-D study

##### Inclusion criteria

1. 18–79 years of age
2. Primary, histologically confirmed resected stage IIb, IIc, IIIa (N1a, N2a) and IIIb (N1a, N2a) cutaneous melanoma
3. Wide excision or, if there is no wide excision, excision of the primary lesion with clear pathological margins <120 days prior to randomisation, with or without sentinel node biopsy
4. Serum corrected calcium and Serum creatinine are ≤1.5 times the institutional upper limit of normal and eGFR within normal range for testing laboratory
5. Serum lactate dehydrogenase < 1.5 upper limit of normal
6. Written informed consent
7. Geographically accessible and willing and able to attend 4 monthly follow-up at MIA for 2 years
8. Performance status ECOG 0–2 (see Appendix 1)

#### Exclusion criteria

1. Patients with a known history of renal calculi.
2. Patients with a known history of hyperparathyroidism.
3. Patients who have a concomitant invasive cancer other than basal cell carcinoma of the skin or localized squamous cell carcinoma of the skin or a previous such cancer and have been cancer free for less than 5 years.
4. Any of the following laboratory test results (tests must not have been carried out more than four weeks prior to randomisation)
  Absolute neutrophil count < 1.5 × 10^9^/L
  Platelet count < 100 × 10^9^/L
  Total bilirubin > 1.5 upper limit of normal
  AST, ALT, Alk Phos > 2.5 upper limit of normal
5. Patients who are pregnant or lactating. Women of child bearing potential must have a confirmed negative pregnancy test at study entry.
6. Patients with a medical or psychosocial problem which, in the investigators opinion, would interfere with treatment or follow-up.
7. Patients with either ocular or mucosal melanoma
8. Patients who are currently enrolled in trials of other experimental treatments or alternative therapies
9. Patients cannot have received any other. investigational agents or treatments (i.e. chemo-, immuno-, vaccine or radiotherapy) within 30 days of commencing study.
10. Patient should not be taking other agents known to interact with the study drug, such as anti-convulsants.

### Appendix 2

#### Safety procedures in the event of abnormal assay results

##### Dose modification

- ■ If serum 25OHD is >200 nmol/l 4–6 weeks after the first dose is given, discontinue all treatment and recommence monthly treatment if and when it falls to <120 nmol/l on 4 monthly monitoring.
- ■ If serum corrected calcium, 24 hour urinary calcium excretion or urinary calcium to creatinine ratio is above the normal range by <20% of the upper limit of normal, discontinue treatment until it returns to within the normal range, then recommence monthly treatment. In the event of temporary discontinuation for either of these reasons, measure 25OHD on stored serum from blood sample collected at the time of the elevated calcium or calcium to creatinine ratio if not a routine 25OHD measurement time.

##### Dose cessation

- ■ eGFR, serum corrected calcium, 24 hour urinary calcium excretion, or calcium to creatinine ratio is >20% above laboratory normal range.
- ■ Development of a renal calculus while on study treatment.
- ■ Pregnancy occurring during the course of the trial.
- ■ Disease progression.

##### Withdrawal because of low serum 25OHD concentration

If a patient is found to have a serum 25OHD concentration of <25 nmol/l at any time it is assayed, he or she will be withdrawn from the trial and offered vitamin D replacement therapy. Because of the desirability of batching vitamin D assays, serum collected at baseline and 4 months will be assayed in a single batch, as will that collected at 12 and 24 months. Neither patients nor their doctors will be told that patients should not take vitamin D supplements during the trial, or have their serum vitamin D concentration assayed if it is clinically indicated. Patients will be asked about use of vitamin D supplements or therapy at each clinic visit.

##### Concomitant medications/treatments

Concomitant medications will not be recorded during the study, except for medications used to treat adverse events or medications known to interact with the study medications.

- ■ Anti-convulsant medication is not allowed.
- ■ Any concomitant use of vitamin D or multi-vitamin supplements in sufficient detail to be able to estimate daily vitamin D intake from these sources.
- ■ Any concomitant use of calcium supplements.

## Authors’ information

RS is a surgical oncologist at Melanoma Institute of Australia (MIA) (formerly the Sydney Melanoma Unit). BA is a public health expert. RM is an expert vitamin D physiologist. RLM is a clinical trialist and executive member of the ANZMTG. KS, AS and JS are surgical oncologists at MIA. JFT is a surgical oncologist, chairman of the ANZMTG and executive director of MIA in Sydney, New South Wales.
